# Upregulated expression levels of ADAM10 and EGFR and downregulated expression levels of E-cadherin in hepatocellular carcinomas

**DOI:** 10.3892/etm.2014.1672

**Published:** 2014-06-03

YUAN YUE, YUMIN YANG, LEI SHI, ZUOREN WANG

Exp Ther Med 6: 1380–1384, 2013; DOI: 10.3892/etm.2013.1340

After the publication of the article, the authors noted that they had made an error regarding certain data in their manuscript.

As the corresponding author of a paper appearing in Experimental and Therapeutic Medicine, I hereby retract the article titled ‘Upregulated expression levels of ADAM10 and EGFR and the down-regulated expression levels of E-cadherin in hepatocellular carcinomas’, published in Exp Ther Med 6: 1380–1384, 2013, because some pictures in this paper came from Dr Shengli Wu’s experiment under the Department of Hepatbiliary Surgery, First Affiliated Hospital of Medical College of Xi’an Jiaotong University. Dr Shengli Wu did not agree with this publication. I apologize for any adverse consequence and inconveniences that may have resulted from the publication of this paper.

